# Supplementary material for: PCSK9 Imperceptibly Affects Chemokine Receptor Expression In Vitro and In Vivo
Source: Int J Mol Sci. 2021 Dec 1;22(23):13026. doi: 10.3390/ijms222313026 (PMC8657700; doi:10.3390/ijms222313026)
Supplement: Supplementary file 1 [file ijms-22-13026-s001.zip › ijms-1455666-supplementary.pdf]

Supplementary Materials

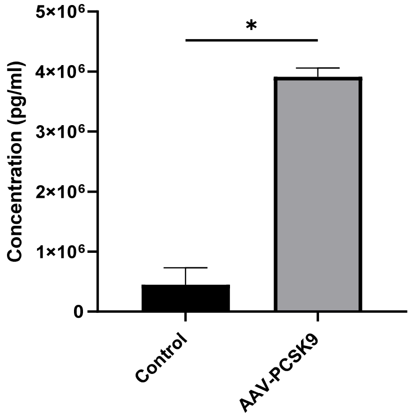

Supplementary Figure S1: PCSK9 levels in C57/BL6 mice. PCSK9 levels in plasma of C57/BL6 mice (n = 8) were checked using PCSK9 ELISA after intravenous injection of AAV-PCSK9. Bar graphs are representation of mean ± SEM. *p<0.05. The statistical difference in the groups were calculated using Mann-Whitney test in GraphPad Prism.

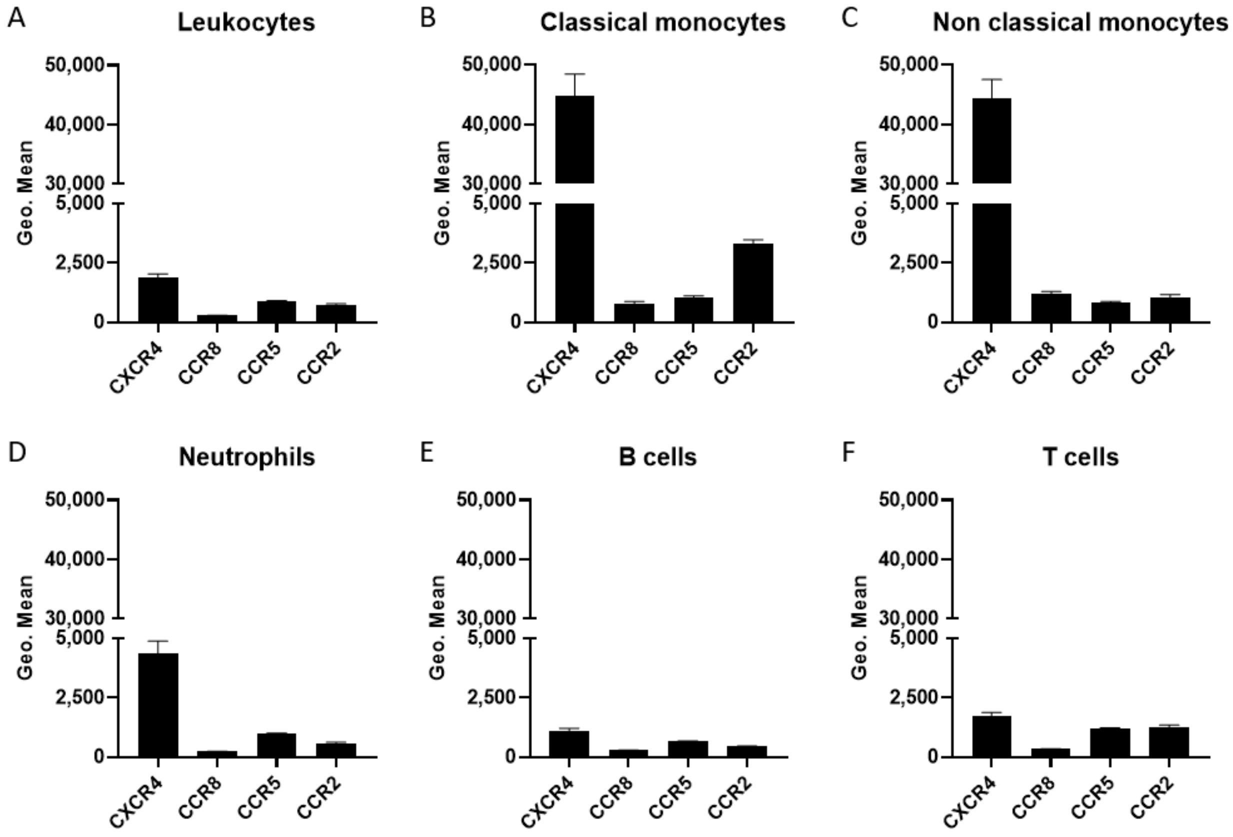

Supplementary Figure S2: Chemokine receptor expression levels in blood leukocytes in C57/BL6 mice. The baseline expression levels of CXCR4, CCR8, CCR5 and CCR2 in leukocytes (A), classical monocytes (B), non-classical monocytes (C), neutrophils (D), B cells (E) and T cells (F) in control mice (n =8) with normal levels of PCSK9, expressed in the form of geometric means. Bar graphs are representation of mean ± SEM.

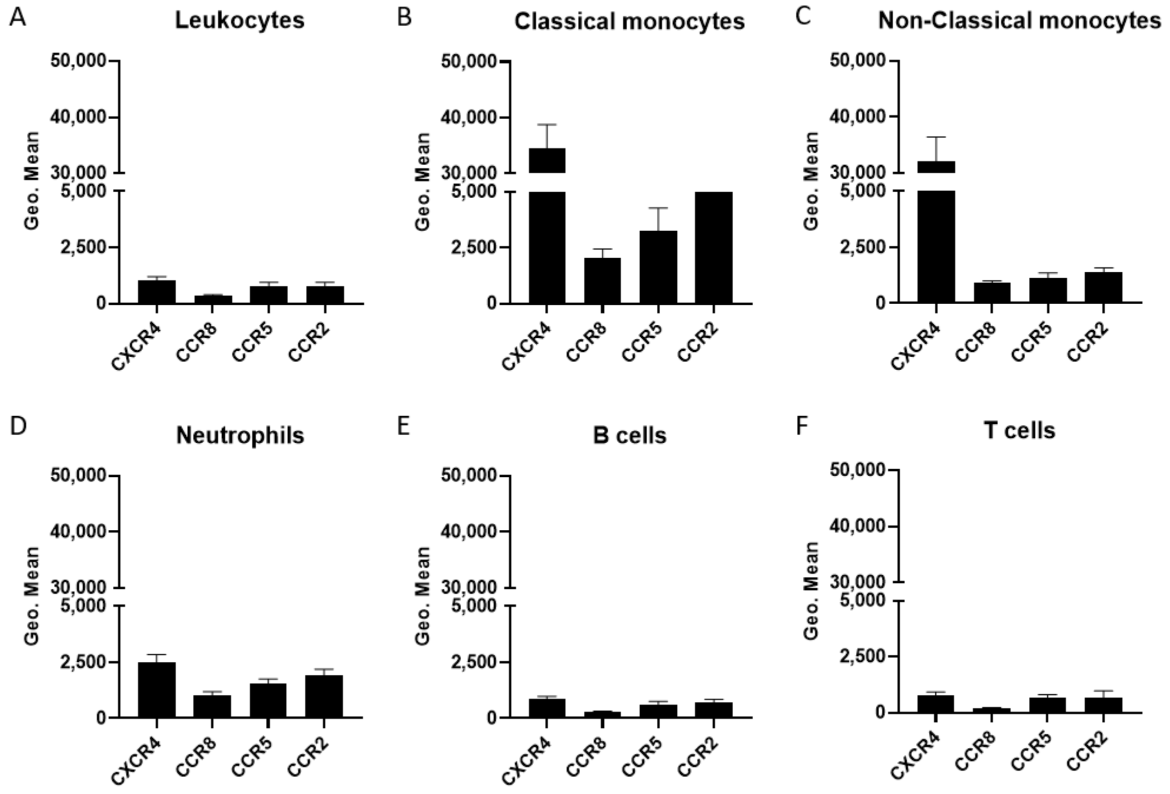

Supplementary Figure S3: Chemokine receptor expression levels in splenic leukocytes in C57/BL6 mice. The baseline expression levels of CXCR4, CCR8, CCR5 and CCR2 in leukocytes (A), classical monocytes (B), non-classical monocytes (C), neutrophils (D), B cells (E) and T cells (F) in control mice (n =8) with normal levels of PCSK9, expressed in the form of geometric means. Bar graphs are representation of mean ± SEM.

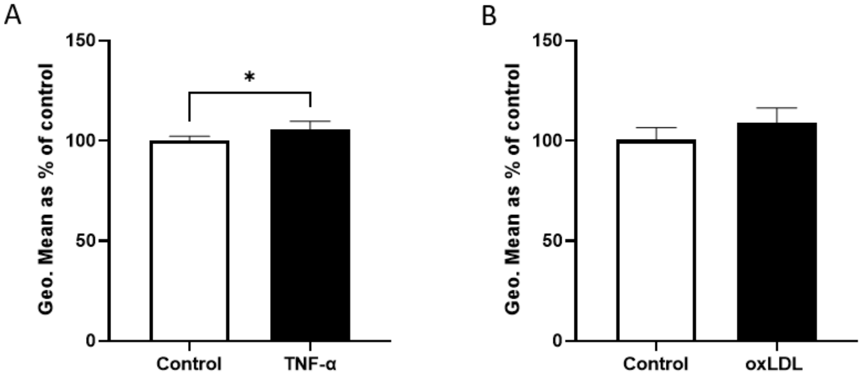

Supplementary Figure S4: CXCR4 expression levels in HCAECs after exposure to Tumor Necrosis Factor-alpha (TNF-α) and oxidized low-density lipoprotein (oxLDL). The expression levels of CXCR4 when HCAECs (n = 2 replicates) were put in an inflammatory environment caused by TNF-α (A) and oxLDL (B), expressed in the form of geometric mean as percentage of control. Bar graphs are representation of mean ± SEM. *$p<0.05$. The statistical difference in the groups were calculated using Mann-Whitney test in GraphPad Prism.

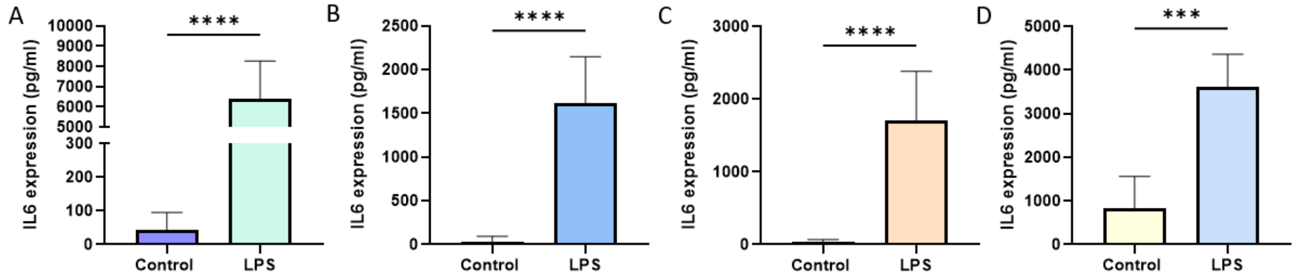

Supplementary Figure S5: IL6 expression levels after stimulation of cells with Lipopolysaccharide (LPS). The expression levels of the inflammatory cytokine IL6 were measured in the supernatant of Bone Marrow Derived Macrophages (BMDMs) (A), human macrophages (B), Human Coronary Artery Endothelial Cells (C) and Human Aortic Smooth Muscle Cells (D) after stimulation with LPS (n = 3-4 replicates). Bar graphs are representation of mean ± SEM. ***$p<0.001$, ****$p<0.0001$. The statistical difference in the groups were calculated using Mann-Whitney test in GraphPad Prism.
